# PD188 Step-By-Step Introduction Of Hospital-Based Health Technology Assessment In Ukraine

**DOI:** 10.1017/S0266462324004100

**Published:** 2025-01-07

**Authors:** Olena Filiniuk, Serhii Siromaha, Anna Dorohina, Bohdan Leskiv, Kostiantyn Yarynych, Kostiantyn Kosyachenko, Mykhailo Babenko, Rabia Sucu

## Abstract

**Introduction:**

Health technology assessment (HTA) became mandatory in Ukraine in 2020 for public coverage of new medicines. This review outlines the steps Ukraine took to introduce HTA at the hospital level through hospital-based HTA (HB-HTA). HB-HTA is a globally recognized, science-based tool that helps hospital stakeholders answer questions on the safety, efficacy, and cost effectiveness of health technologies they are planning to introduce.

**Methods:**

The authors examined the steps taken to introduce and implement HB-HTA in Ukraine, the impacts reached thus far, and the implications for further national scale-up. Key steps associated with the intervention included the following: awareness raising activities in HB-HTA; structured stakeholder engagement; review of global HB-HTA tools and their adaptation to the local context; training for key hospital staff; piloting tools in a select hospital; and review of the pilot experience to inform future application.

**Results:**

Starting from 2021, HB-HTA was introduced through awareness raising activities. The regulatory framework for introducing health technologies into hospitals was analyzed, outlining the need for updated regulations. Review of global HB-HTA tools revealed that tools developed by the Adopting Hospital Based Health Technology Assessment in European Union project were the most applicable for Ukraine and were subsequently adapted to the country’s context by an expert panel of 13 hospital stakeholders. Following this, Safe, Affordable, and Effective Medicines for Ukrainians (SAFEMed) organized HB-HTA training for key hospital stakeholders. The Amosov National Institute of Cardiovascular Surgery was chosen for the pilot due to its high level of operational activity. The results of the pilot are expected soon.

**Conclusions:**

Introducing globally recognized HB-HTA tools and piloting them in a real-world setting provides basic concepts to the HTA experts who will be involved in scaling up HB-HTA in Ukraine. Initial recommendations point to the need to scale-up HB-HTA slowly, first working with one hospital to gain HB-HTA experience that will inform further application across the country.

